# Improvement of Warm-Mix Asphalt Concrete Performance with Lignin Obtained from Bioethanol Production from Forest Biomass Waste

**DOI:** 10.3390/ma16237339

**Published:** 2023-11-25

**Authors:** André Pascoal, Arminda Almeida, Silvino Capitão, Luís Picado-Santos

**Affiliations:** 1 Departamento de Engenharia Civil, Universidade de Coimbra, Rua Luís Reis Santos, 3030-788 Coimbra, Portugal; andrepascoal@uc.pt (A.P.); arminda@dec.uc.pt (A.A.); 2 CITTA—Centro de Investigação do Território, Transportes e Ambiente, Rua Dr. Roberto Frias, 4200-465 Porto, Portugal; 3 Instituto Superior de Engenharia de Coimbra, Instituto Politécnico de Coimbra, SusCita, Rua Pedro Nunes, 3030-199 Coimbra, Portugal; 4 CERIS—Civil Engineering Research and Innovation for Sustainability, Instituto Superior Técnico, Universidade de Lisboa, Av. Rovisco Pais, 1049-001 Lisboa, Portugal; luispicadosantos@tecnico.ulisboa.pt

**Keywords:** adhesion, asphalt additive, circular economy, lignin, sustainability

## Abstract

This study aims to assess the effect of adding lignin waste, a by-product of bioethanol production from forest biomass, to asphalt concrete to improve its performance. After adjusting the lignin content based on preliminary Marshall tests, 20% of this by-product by mass of bitumen was added to the asphalt concrete blends via the dry method. This lignin content was suitable to the temperature was decreased 40 °C compared to the usual mixing temperature, thus allowing the production of warm-mix asphalt concrete (WMA) without any other additive. Tests on a gyratory compactor assessed the workability of the studied asphalt concrete, allowing us to obtain these findings. Moreover, lignin improved moisture damage and adhesion resistance between the binder film and the aggregate particles’ surface. The behaviour at high temperatures was also enhanced, resulting in better resistance to permanent deformation. These promising laboratory results show us an opportunity to create value for this type of by-product in substituting commercial additives for asphalt concrete, such as organic wax or adhesion promoters, to allow the production of warm-mix asphalt concrete with improved properties.

## 1. Introduction

### 1.1. Background

The world’s energy demand still relies significantly on fossil fuels, which have an 80% share [1]. The scenario has been changing, but much more slowly than is desirable [1]. According to the European Environment Agency, the implemented policies increased the use of renewable energy, reducing the use of petroleum-based products. Considering the strategy and priorities of the developed economies, namely in the European Union, decarbonisation in the transport sector encompasses increasing the use of electric vehicles and the consumption of biofuels [2], whose global use is expected to grow. In addition, the revised Renewable Energy Directive (RED II) [3] introduced sustainable criteria in biofuel production to mitigate its negative impact on indirect land use. Environmental and economic concerns have led to the application of lignocellulosic biomass products in asphalt modification (both asphalt binder and mixture modification). Despite being an abundant and inexpensive material, its cost-effectiveness depends on the techniques used to valorise it [4]. Lignocellulosic biomass products—resulting from pyrolysis processes—include lignin, cellulose, hemicellulose, biochar, and bio-oil [4,5]. Given the nature of lignocellulosic biomass, some materials can pose issues regarding the durability of asphalt concrete [6,7] since they can be susceptible to degradation over time. However, their degradation resistance can be enhanced using chemical treatments or coating methods [8]. In asphalt concrete, those materials are coated by bitumen; consequently, their degradation exposure is minimised. Those products have been subjected to intense research by the scientific community and the pavement industry, mainly regarding asphalt binder modification [9]. It should be noted that the residues’ source, content, and valorisation techniques used influence the results. Ataeian et al. [10] reviewed the binder’s nano-cellulose modification, concluding that it can improve ageing, fatigue, and rutting resistance. Cellulose fibres have also been used in asphalt concrete modification [11].

Portugal, for instance, has a forestry plan that supports the reforestation of burned areas, the cleaning of forests (waste), and the paper industry (which produces bark wastes). These activities generate large amounts of lignocellulosic biomass waste suitable for biofuel (ethanol, for instance). Although eliminating lignin is essential in producing biofuels when utilising lignocellulosic materials, it remains a crucial feedstock for generating a sustainable and renewable energy source presently and in the future [12]. The process generates roughly three parts of lignin as a by-product for one part of ethanol produced from that feedstock. While lignin content depends on plant categories and their conservation conditions, the highest is found in hardwood trees, some bushes, and perennial grasses. Therefore, the transition towards transport decarbonisation may be complemented by valuing lignin in transport infrastructure’s pavements as a partial bitumen substitute or extender/modifier. Moreover, using that by-product as feedstock to produce bio-binders will contribute to the competitiveness of the European Union’s regions by substituting part of petroleum-based fuels and binders with native raw materials.

### 1.2. Previous Findings on the Use of Lignin in Asphalt and Contributions of This Study

Although the use of lignin as pavement material is not recent, the interest in more profound knowledge on the capabilities of lignin to be used as a sustainable constituent of binders and asphalt concrete has recently increased [13]. Gaudenzi et al. [13] published a review on using lignin from different sources and processes as raw material for sustainable pavements. Different sources and extraction processes may lead to diverse lignin properties [14], preventing generalising findings from published studies [13]. Lignin is one of the planet’s most abundant biopolymers (large molecules composed of repeating structural units) that provide structural support and rigidity to different types of plants. It is a hydrocarbon formed by a three-dimensional network of compounds similar to bitumen’s resins and asphaltenes’ fractions [15,16]. Obtaining the chemical composition of lignin is challenging due to structural differences between lignin from various species [17]. Nevertheless, the three main monomeric units of lignin, also named building blocks, mentioned in the literature are coniferyl alcohol, sinapyl alcohol, and p-coumaryl alcohol [18]. Most studies regarding the use of lignin for asphalt pavements involve dispersing lignin into bitumen to produce bio-binders via different protocols [13], aiming to achieve a certain level of interaction between the materials. This so-called “wet process” changes the asphalt binder’s physical and/or chemical properties. Some authors [19] suggest that adding lignin during mixing to manufacture asphalt concrete (“dry process”) instead of adding it to bitumen should not be applied if the objective is to use lignin as a filler replacement because part of the additive will act as a binder.

Bitumen with lignin is generally obtained by mixing lignin (in liquid or powder form) with bitumen at high temperatures (150–180 °C) using a shear mixer rotating at 4000–6000 rpm for a specific time (generally, 30–60 min). The quantity of lignin blended with bitumen has been reported to vary from 5 to 30% by weight of bitumen. The resulting product may be considered a bio-bitumen as it incorporates lignin, a biomaterial [20,21,22,23,24]. The wet process has been applied, aiming to improve the asphalt binder’s properties. The enhancement of ageing resistance [25,26,27] and the change in viscosity [22,28] are consistently recognised in the literature. The analysis of the resulting bio-bitumen reveals that the hardening increases as the percentage of lignin increases [25], resulting in lower penetration and higher softening point and viscosity [13]. As a result, workability issues may arise when the lignin content is too high (higher than 20%) or when using a higher-viscosity base bitumen [29]. Although the literature states no chemical reaction exists between lignin and bitumen, some positive contributions are reported, such as improving ageing resistance due to oxidative hardening [14,21,30]. The micro-scale analysis also shows that adhesion between the lignin binder and the aggregate surface is enhanced compared to virgin bitumen [14,25]. Ghabchi [14] concluded that adding 20% of any three lignin wastes to a Superpave PG 82-28 bitumen improved the adhesion of the resulting bio-binder to quartzite and granite aggregates. These findings were obtained in pull-off strength tests for dry and wet conditions.

The literature has also reported considerable improvements in the binder’s rheology, such as enhanced resistance to permanent deformation at high temperatures of bio-bitumen with lignin [14,21]. Results for the complex shear modulus (G*) and phase angle (δ) obtained from the dynamic shear rheometer (DSR) test conducted at different temperatures resulted in increased rutting factor (G*/sin δ) values for unaged and RTFO-aged (rolling-thin film oven test) samples when 20% of lignin was added to bitumen. Although the rutting factor decreased as temperature increased from 52 °C to 70 °C, the bio-binders made with different types of enzymatic hydrolysis lignin (the use of enzymes to break down lignin) were consistently higher, showing a higher rutting resistance of the bio-binders [14]. Other authors [22] confirmed this improved resistance to rutting for different types and lignin contents, although the rutting resistance of the bio-bitumen with lignin varies with the lignin type. The hardening resulting from lignin is likely to slightly decrease the resistance of bio-binder against fatigue at intermediate temperatures [21,22]. These findings have been observed by determining the Superpave fatigue parameter (G* sin δ) determined in the DSR test at intermediate temperature (around 30 °C), revealing small increments of G* sin δ and therefore inferior fatigue resistance for the bio-bitumen with lignin compared to the results of the base bitumen. These results were confirmed in Linear Amplitude Sweep tests (LAS) conducted in PAV-aged samples in which the number of cycles to fatigue was slightly lower for the bio-binder than the base bitumen [22]. Norgbey et al. [29] analysed the storage stability of bio-binder produced by the wet process and concluded that a significant difference occurred for the binder’s penetration at 25 °C and complex modulus in the range of 30 °C to 80 °C. These results allow us to anticipate that heated tanks with agitation will probably be required to ensure the storage homogeneity of bio-binder in industrial production.

Although there have been few studies on the performance of asphalt concrete with lignin [13], some tendencies have been reported considering lignin obtained from different sources and processes. Marshall stability (strength) is generally higher, and Marshall flow (deformation) is lower [31]. The observed improved behaviour for the bio-bitumen is confirmed for permanent deformation resistance [32] and adhesion between the binder and the aggregate surface [24]. Moreover, the stiffening influence of lignin has also been observed in asphalt concrete, which revealed a higher resilient modulus [24,31]. Improved resistance against moisture [22,24] and fatigue has also been reported [31].

Although many laboratory studies have found that there are significant potentialities of bio-bitumen resulting from mixing lignin and bitumen via the wet process to improve the properties of hot asphalt concrete, further laboratory analysis is needed to assess if some of those expectations can be observed directly in asphalt concrete manufactured with lignin added by the dry process. Specifically, in the case of warm-mix asphalt concrete manufactured at temperatures 30 °C to 40 °C lower than conventional hot-mix asphalt concrete, the paper’s goal is to evaluate the capability of lignin to replace part of the bitumen and the expensive Fisher–Tropsch organic wax used as an additive to allow a reduced manufacturing temperature compared to hot-mix asphalt concrete. Because the wax has a melting point between 90 °C and 100 °C, it is usually used to reduce the binder’s viscosity to reach suitable mixing and compaction conditions of the asphalt concrete. Therefore, the wax replacement by lignin requires further study because the workability of the asphalt concrete may be an issue. Moreover, this study also contributes to assessing if lignin obtained in the production of biofuel (ethanol) from the lignocellulosic biomass waste can reduce some reported possible weaknesses of warm-mix asphalt concrete [15], such as water sensitivity, bitumen–aggregate adhesion, and rutting resistance.

## 2. Materials and Methods

### 2.1. Aggregates, Bitumen, and Organic Wax

**Figure 1:**
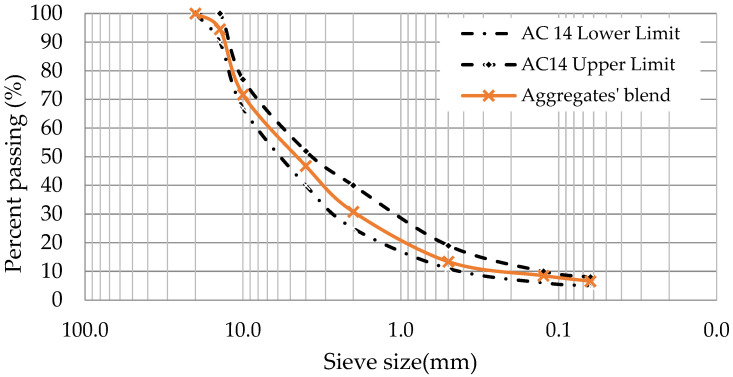
Aggregates’ blend and Portuguese road administration’s limits.

The aggregates’ blend has two fractions of crushed gneiss particles (8/20 and 4/12 mm) and two fractions of limestone particles (0/4 mm and filler). This fulfils the Portuguese road administration’s specifications regarding granulometry [33]. Figure 1 shows the gradation of the studied dense-graded asphalt concrete (AC) for the surface layer—AC 14 surf 35/50 [33] and the gradation limits. For ease of analysis, Table 1 is reproduced from a previous study published by the authors [34,35] containing the used aggregates’ physical properties and the Portuguese specifications’ requirements considered in EN 13043 [36].

The asphalt concrete blends were produced with a conventional 35/50 paving grade bitumen, with penetration at 25 °C of 45 0.01 mm and a softening point of 52 °C. The AC has a well-established bitumen content of 5.0% by mass of the total mixture.

This study uses an organic wax, commercially known as Sasobit-Redux^®^ (Sasol, Hamburg, Germany), as an additive to manufacture and compact the conventional warm-mix asphalt concrete (WMA) at low temperature levels [45]. This product is soluble in bitumen at temperatures above 85 °C. Following the supplier’s recommendations, 1.5% wax (by weight of bitumen) was considered. The additive was added to the blend during the mixing process.

### 2.2. Lignin

**Figure 2:**
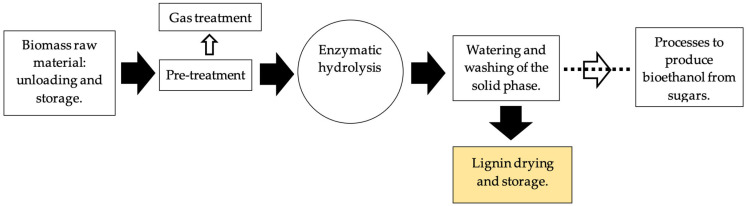
Process framework to produce the used lignin.

The company Bioadvance—The Next Generation, Lda (Guia, Portugal) supplied the lignin considered for this study. It is a by-product of a proprietary bioethanol production process from forest biomass, such as wood chips, sawdust, and bark, prepared for processing by reducing the feedstock size. This processing allows further pre-treatment to be carried out to break down the existing lignocellulosic structure, making it easier for lignin recovery. Figure 2 shows a simplified process framework to obtain the used lignin.

The forest biomass that arrives at the unit is unloaded, stored, and subjected to screening and other operations to separate inappropriate raw materials before the feedstock proceeds to the pre-treatment phase. These operations aim to open the lignocellulosic material’s structure, making it reachable for enzymatic hydrolysis. This material is impregnated with SO_2_ as a catalyst before entering a reactor in which the lignocellulosic material is subjected to specific pressure and temperature conditions for a defined time. The pre-treatment phase separates the steam from the obtained biomass slurry, which is then diluted with water in a separate tank before entering the enzymatic hydrolysis process. The enzymatic hydrolysis converts the cellulosic solid material into monomeric sugars by using enzymes when needed in a temperature range of 45–55 °C for a predefined time. The obtained solid phase is subjected to dewatering and washing to separate the lasting insoluble solids from the liquids and collect as much sugar as possible in the liquid fraction. Then, the solid material rich in lignin is filtered to remove moisture, washed with clean water, and blown to dry from approximately 50% to 5% of moisture content. Depending on the feedstock used, the supplier declares that the chemical composition is 65–70% carbon, 30–35% oxygen, up to 5% hydrogen, and around 0.01% sulphur.

**Figure 3:**
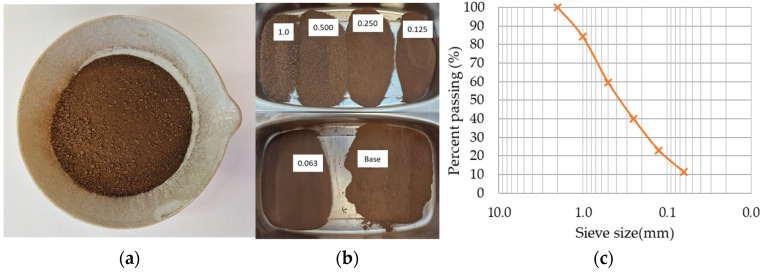
Lignin: (**a**) view of lignin powder, (**b**) material retained in each sieve, (**c**) lignin size distribution.

The used lignin is a fine, brown powder with 5.7% water content, with the aspect and gradation shown in Figure 3. This material is a sulphur-free additive with the main components obtained by the supplier after enzymatic hydrolysis and liquid phase separation presented in Table 2.

The two main solid lignin components are glucan and Klason lignin. Less than 0.5% of several other components, such as arabinan, galactan, xylan, and mannan were also identified. Glucan is a polysaccharide found in the cell walls of plants, playing an important structural role. Klason lignin is the acid-insoluble lignin—the fraction of lignin left after the removal of cellulose and hemicellulose. It represents a measure of the lignin content in a sample of biomass.

### 2.3. Blends Compositions

The adequate lignin content was established by testing specimens prepared with 5% lignin increases by mass of the reference bitumen in the Marshall compression machine. It must be emphasised that as the lignin content increased, the bitumen content decreased accordingly to obtain a constant bitumen + lignin content of 5% by mass of the total blend.

In order to analyse the improvement of the warm-mix asphalt concrete performance with lignin, some of the results were compared with a WMA with 1.5% organic wax by mass of total bitumen.

### 2.4. Methods

The applied methods aim to evaluate the performance of a WMA with lignin and compare it to a hot-mix asphalt concrete (HMA). The WMA was mixed at 130 °C, while the HMA was mixed at 170 °C. The lignin amount was dried in an oven at 105 °C and added to the mixing bowl (dry process) during the manufacturing procedure of the WMA. The performance of a WMA with wax was also evaluated for comparison with mixtures without lignin. For the WMA with wax, 1.5% of the additive by weight of bitumen was previously added to the bitumen and stirred until dissolved. Then, depending on the test to be carried out, specimens were prepared using the impact [46], gyratory [47], or roller compactors [48].

**Figure 4:**
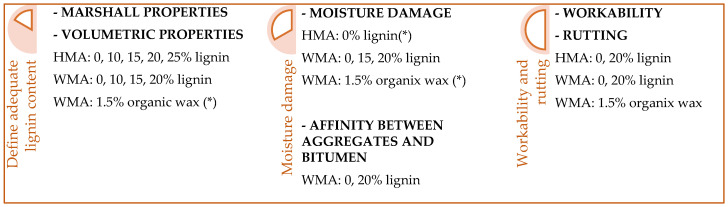
Phases of the study. (*) Results from previous authors’ research [35].

First, Marshall tests and volumetric properties evaluation supported determining adequate lignin content. In the following stages, tests were conducted to evaluate WMA’s main potential drawbacks (moisture damage, workability and rutting resistance) [49,50]. Figure 4 depicts the phases of the study and the corresponding tested mixture. Some results of HMA and WMA with wax came from previous research performed by the authors [35].

Afterwards, as the costs of developing alternative technologies or materials should not be disregarded, a direct cost analysis for each asphalt concrete was conducted to evaluate the probable savings.

#### 2.4.1. Marshall and Volumetric Properties

The Marshall and volumetric properties were used to define the adequate lignin content. For that, mixtures with 0%, 5%, 10%, 15%, 20%, and 25% of lignin by bitumen mass were considered, and four cylindrical specimens per mixture were prepared (impact compactor, 75 blows per face). Once the specimens were prepared, their density was determined (saturated surface dry) [51] before being subjected to the Marshall test [52] at 60 °C. The maximum density of each mixture was also evaluated [53] to determine void content [54].

#### 2.4.2. Moisture Damage

Moisture damage can shorten pavement life [55], particularly for pavements with WMA, since the low-production temperature cannot fully dry the aggregates and water may exist in the aggregated micropores [55]. It is thus crucial to assess how the bond between the bitumen and the aggregates behaves in the presence of water. Several tests (qualitative and quantitative measures) have been developed on loose and compacted mixtures [55,56]. This study uses two tests to assess moisture damage of the WMA with and without lignin. The rolling bottle test is a qualitative one applied over the loose mixture [57], and the indirect tensile strength ratio (ITSR) is a quantitative parameter obtained on compacted specimens [58].

The rolling bottle test aims to determine the affinity between aggregates and bitumen by visually registering the bitumen coverage on aggregate particles after mechanical stirring action in the presence of water. For that, three samples per mixture were prepared at 130 °C. One mixture contains 0% lignin, while the other contains 20%. Each sample was transferred to each bottle, and distilled water was added to fill it until it was shoulder-level. Then, the glass rods were placed in the bottle, and the bottles were sealed with screw caps and placed on the rolling machine (rotating at 60 revs min^−1^). The rolling procedure started, and the bitumen coverage on aggregate particles was evaluated at 6 h, 24 h, 48 h, and 72 h of rolling time. As the visual estimation of the degree of bitumen coverage can be the major drawback of this test [59], a photo of each sample was taken, and the Image Processing Toolbox of the Matlab program (Matlab R2023a) was used to process them. It is noted that every photo was taken in the same conditions. First, the Color Threshold App was applied to turn the photo into a binary image, and then the Image Region Analyzer App was used to calculate the bitumen coverage area.

To determine the ITSR, eight cylindrical specimens per mix were prepared (impact compactor, 50 blows per face), and then the set was divided into two subsets. One subset is maintained dry at room temperature, while the other is conditioned in water. After conditioning, each specimen’s indirect tensile strength (ITS) was determined at the test temperature (15 °C) [60]. The ITSR value is the ratio between the ITS of water-conditioned specimens and the dry specimens.

#### 2.4.3. Workability

Adequate compaction is crucial for improved pavement performance [61,62]. Compaction reduces the mixture’s air void content due to rearranging aggregate skeleton structure. The energy applied in the compaction process permits evaluating workability.

WMA are mixtures produced at temperatures lower than conventional HMA; in this study, they were produced at 40 °C below. Although these mixtures incorporate additives to improve workability, it is essential to evaluate them since mixing temperature influences bitumen viscosity and thus workability.

**Figure 5:**
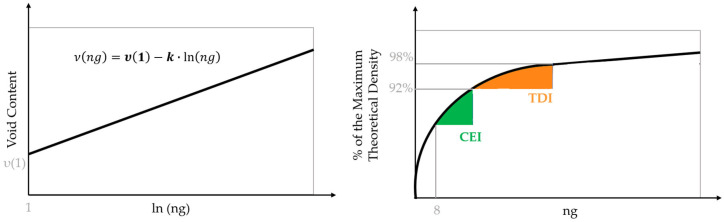
Workability parameters (ng: number of gyrations).

Several parameters have been developed to measure workability [63,64]. The developed parameters have different intentions, as compaction occurs during paving construction (paver screed and rollers) and in in-service pavement (traffic densification). Most are based on the compaction curves obtained in the gyratory compactor, for which the number of gyrations characterises the compaction energy. It applies a compressive load (600 kPa) with a slight vertical deviation angle (1.16°) to better reproduce how the mixture is compacted in the field. The European Standard EN 12697-10 [65] fits a linear equation to the relationship between the air void content and the logarithm of the number of gyrations (compaction curve) and uses the the parameters ν(1)—void content for one gyration—and k—compactability—to measure workability. Faheem and Bahia [63] proposed the Compaction Energy Index (CEI) and Traffic Densification Index (TDI) to evaluate mixture resistance to compaction during pavement construction and densification under traffic, respectively. CEI is the area under the compaction curve between the eighth gyration and the point corresponding to a density of 92% of the maximum theoretical one. In turn, TDI is defined as the area under the compaction curve between the points corresponding to densities of 92% and 98% of its maximum theoretical value. Figure 5 illustrates the workability parameters. Researchers have often used these parameters [65,66,67] to evaluate workability.

The ν(1) indicates the specimen density after the first gyration of the compactor. Thus, a mixture with a higher value presents a higher workability. The slope of the semi-logarithmic linear equation, k, indicates how fast the compaction is, so mixtures with higher k values indicate better compactability. The CEI indicates the energy required in the construction period, i.e., the energy required for the mixture to achieve the desired density; mixtures with higher CEI values present lower workability. Finally, the TDI indicates how stable the mixture is during traffic densification (pavement in service); a more stable mixture presents a higher TDI value. This paper presents three of the four parameters as specimen densities at the end of the gyratory compactor have not achieved 98% of the maximum.

#### 2.4.4. Rutting

The wheel-tracking test (small size device, procedure B in air) was used in this study to evaluate the susceptibility of the mixture to deform under load (rutting resistance) at a high temperature, 60 °C [68]. Two specimens per mixture (with 40 mm thickness) were prepared in the roller compactor and tested after temperature conditioning. During the test, the rut depth formed on the slab surface by the repeated passes of a loaded wheel is recorded. The wheel passes for 10,000 cycles, or fewer if a ruth depth of 20 mm is reached first. At the end of the test, the following parameters are calculated: the ruth depth (RD_AIR_), the wheel-tracking slope (WTS_AIR_) and the proportional rut depth (PRD_AIR_).

## 3. Results and Discussion

### 3.1. Marshall and Volumetric Properties

**Figure 6:**
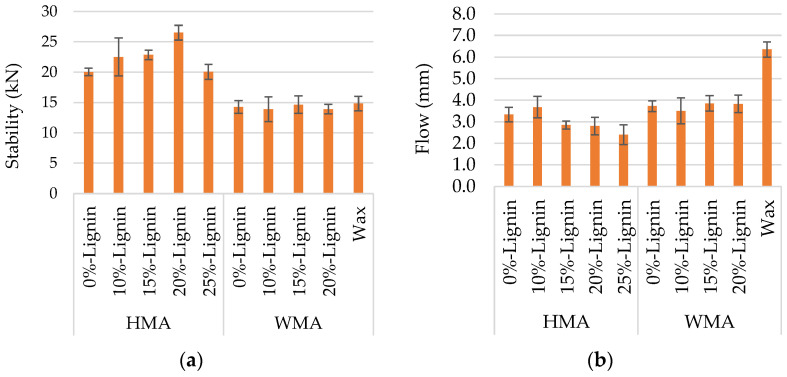
Marshall properties. (**a**) stability; (**b**) flow.

**Figure 7:**
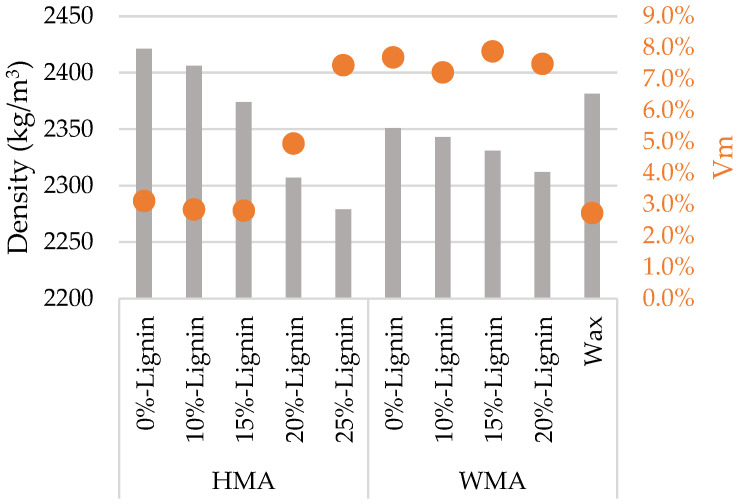
Volumetric properties.

As mentioned above, Marshall and volumetric properties defined the adequate lignin content. Figure 6 shows that lignin content influences the stability of HMA more than the stability of WMA. The highest stability value was achieved for 20% of lignin, which was the most adequate. Looking for volumetric properties (Figure 7), WMA mixtures incorporating lignin tend to present a higher air void content, which does not depend substantially on the lignin content. The mixture with wax appears to stand out; however, it is noted that the result comes from other authors’ research [36], in which different operators and devices were used. The vertical error bar in each figure represents the standard deviation of four specimens.

### 3.2. Moisture Damage

#### 3.2.1. Rolling Bottle Test

**Figure 8:**
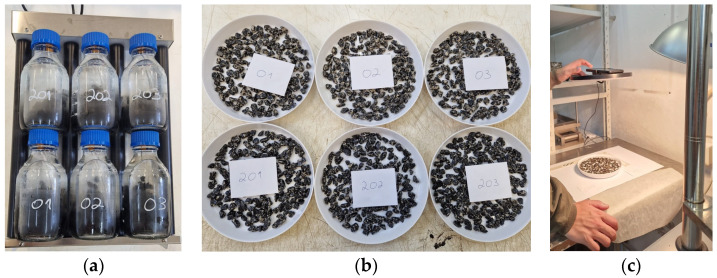
Rolling bottle test: (**a**) bottles on the rolling machine; (**b**) image of the six samples after 6 h of rolling time; (**c**) photo-taking process.

**Figure 9:**
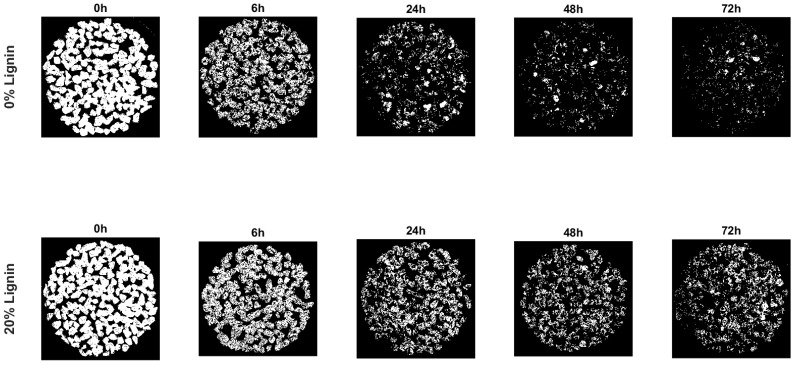
Color Thresholder App—binary images.

**Figure 10:**
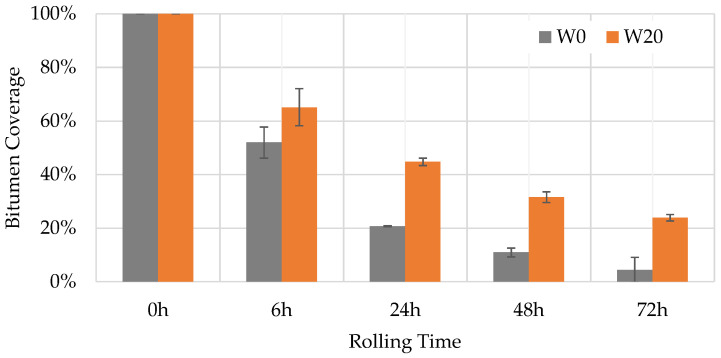
Reduction in bitumen coverage with rolling time.

Figure 8, Figure 9 and Figure 10 show some test phases, the use of Matlab’s (Matlab R2023a) Image Processing Toolbox, and the percentage of the bitumen coverage on the aggregates during the rolling time, respectively. Two mixtures were assessed: a WMA without lignin and a WMA with 20% lignin.

The Color Thresholder App was used to convert the taken photo into a binary image in which white represents bitumen coverage. Figure 9 shows the binary image for two samples, one with 0% lignin and the other with 20%.

The Image Region Analyzer App was used to quantify the binary images’ white areas. Figure 10 shows the average values of the three bottles and the corresponding standard deviation values. After mixture preparation (rolling time equal to zero), the bitumen coverage is 100%, corresponding to a white area. Then, with the rolling time, the bitumen coverage decreases, and consequently, the white area does as well. The percentage of bitumen coverage represents the white area in each rolling time concerning the area after sample preparation.

The incorporation of lignin resulted in a higher bitumen coverage (+20%) at the end of the test. It enhances the affinity between the bitumen and the aggregates, which aligns with other researchers’ results [29,69]. The water sensitivity of compacted specimens is analysed in the next section for better knowledge of moisture damage, comparing results for HMA and WMA with wax.

#### 3.2.2. Indirect Tensile Strength Ratio (ITSR)

**Figure 11:**
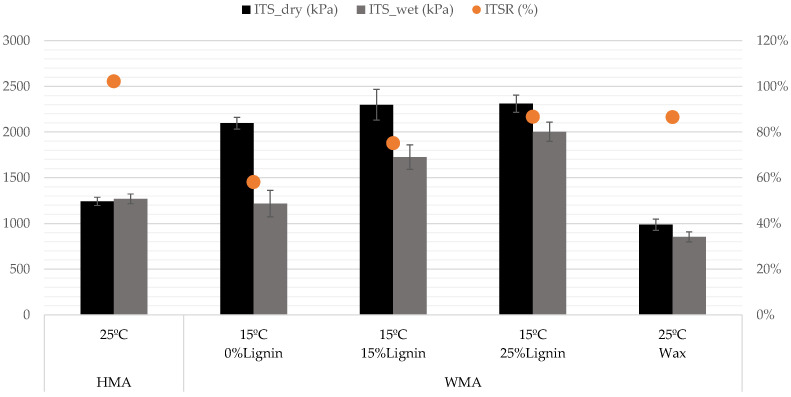
ITS of water-conditioned specimens and the dry specimens and ITSR.

Figure 11 shows ITS (indirect tensile strength) values of water-conditioned specimens, dry specimens, and ITSR. The vertical error bar represents the standard deviation of four specimens. The results of the HMA (0% lignin) and WMA with wax are from earlier authors’ research [35]. The corresponding values are lower because the test temperature was 25 °C instead of the 15 °C used in this study. At lower test temperatures, the ITS values are higher. Even so, the results of ITSR at both temperatures provide comparable measures for the water sensitivity of asphalt concrete blends.

From the figure, it can be observed that increasing lignin content makes the WMA less sensitive to water. From 0% lignin to 20% lignin, the ITSR value increased by 29%, leaving the WMA with 20% lignin up from the threshold of 80% usually considered by the Road Administrations and a water sensitivity similar to the WMA with wax.

The production temperature greatly influenced the water sensitivity. When comparing the HMA and WMA results (without lignin), a temperature reduction of 40 °C led to an ITSR reduction of 44%. To some degree, the decrease can be explained by the higher WMA’s air void content (8% compared to 3%) and the possible presence of water in the aggregates. Adding lignin reduced the air void content by 2% and promoted adhesion between the aggregates and bitumen, making the mixture more resistant to moisture damage, proving the use of lignin in improving moisture damage resistance.

### 3.3. Workability

**Figure 12:**
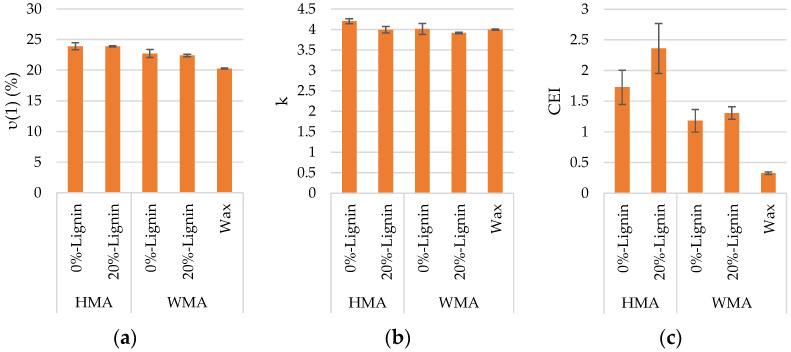
Workability parameters for the tested mixtures: (**a**) υ(1), void content for one gyration; (**b**) compactability; and (**c**) Compaction Energy Index (CEI).

Figure 12 presents the workability parameters. The vertical error bar represents the standard deviation of three specimens.

Although the parameters ν(1) and k were not significantly influenced by the incorporation of lignin in both HMA and WMA, the latter revealed a slightly better compactability with 20% lignin. Concerning the CEI parameter, there are differences between mixtures. Since mixtures with lower CEI values present better workability, incorporating lignin into the WMA improved its workability. On the contrary, HMA exhibits slightly higher resistance to compaction with 20% lignin.

It should be noted that the k parameter fits the entire densification curve from the first to the last cycle (120 gyrations). In contrast, the CEI parameter refers only to gyrations between the eighth gyration and the point corresponding to a density of 92% of the maximum theoretical value. Comparing the parameters k and CEI, the results show that CEI is more sensitive to the incorporation of lignin than k. Overall, it can be stated that lignin does not pose workability problems even in warm-mix asphalt concrete produced at 40 °C below HMA.

### 3.4. Rutting Resistance

**Figure 13:**
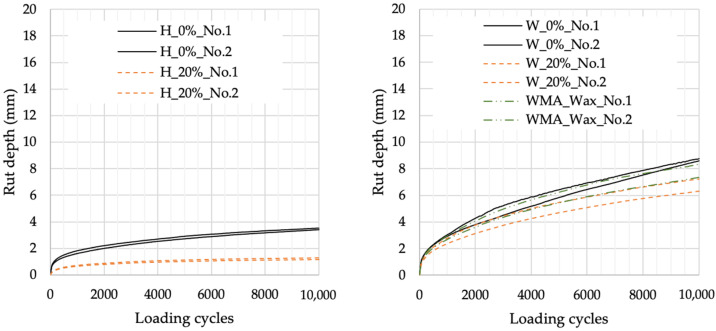
Rut depth evolution with loading cycles.

Figure 13 presents the evolution of rut depth with loading cycles, and Table 3 summarises the parameters obtained in the wheel tracking test. As can be observed, the incorporation of lignin improves the rutting resistance of HMA and WMA. The WMA with 20% lignin performs even better than the WMA with wax. Lignin improves the mechanical response of the mixture to plastic deformation.

### 3.5. Direct Cost Analysis

From a contractor’s perspective, direct costs play a pivotal role, as they are a critical aspect under scrutiny when determining the feasibility of using alternative technologies or materials in producing asphalt concrete. Nonetheless, attraction to contractors depends on several primary factors: the cost associated with the raw materials and the production expenses involved in creating asphalt concrete with alternative raw materials alongside the asphalt layer’s long-term durability and performance throughout the contract’s warranty period.

Consequently, the direct costs associated with typical hot- and warm-mix asphalt concrete and warm-mix asphalt concrete with lignin as an additive were examined to evaluate the probable savings. This analysis was conducted within the context of the Portuguese construction environment, accounting for factors like the average procurement expenses for raw materials, equipment, and labour costs and the standard profit margins commonly considered in public construction projects. Table 4 summarises the calculation of the average direct costs for asphalt concrete produced using the components detailed in the table. The simulations considered certain assumptions related to transportation, such as if aggregate transportation costs (from quarries or processing facilities to asphalt plants) were based on an average transport distance of 20 kilometres.

The results reveal that the WMA with lignin as an additive has lower direct costs than conventional HMA and WMA produced with organic wax. Compared to a conventional WMA with wax, the direct costs may be reduced by 12.5% for WMA with lignin because there are savings associated with bitumen and wax replacement. Considering the expectations on further durability based on the mechanical performance of WMA with lignin of around 20% and bearing in mind the better binder adhesion to aggregates and rutting resistance, these savings may roughly reach 30%. These expectations should be confirmed after the complementary laboratory and field experiments.

## 4. Conclusions

The study presented in this paper showcases the benefits achieved in warm-mix asphalt concrete properties when adding lignin obtained from forest waste biomass in ethanol production. The findings also confirm that some potentialities mentioned in the literature for bio-bitumen with lignin are observed for warm-mix asphalt concrete with lignin waste added by the dry process as a substitute for organic wax and part of the bitumen.

The results obtained from the study allow us to summarise the following conclusions:

- Marshall stability increased as lignin content increased up to 20% for HMA, but the influence was not visible for the WMA, and 15 or 20% lignin reduced Marshall flow for HMA and slightly increased it for WMA.
- The void content seems not to be considerably influenced by adding lignin and reducing bitumen contents for WMA.
- Adding lignin by the dry process enhanced the affinity between the bitumen and aggregates, comparing traditional WMA with wax and WMA with lignin.
- Water sensitivity was not an issue for the 20% lignin WMA blend, which achieved ITSR values above 80%. Despite the reduction in bitumen content, ITSR achieved good performance, showing that lignin compensated for the effect of the replaced bitumen.
- Although the different parameters used to assess workability delivered dissimilar conclusions, the findings show that the workability of WMA is not negatively influenced by 20% lignin. CEI results indicate improvement of WMA’s resistance to compaction despite this not occurring for HMA.
- Addition of 20% lignin improved the rutting resistance performances of HMA and WMA. The most significant outcome is that the resistance was better than that observed for the WMA with wax (without lignin).

Because the results obtained are promising, future work will be carried out on this WMA to assess stiffness, phase angles, and fatigue resistance. Moreover, ageing may be an issue and, therefore, accelerated ageing in TEAGE [70] will simulate several years of exposure to moisture and solar radiation to understand if the potential weaknesses concerning ageing and fatigue will occur after ageing. In addition, pavement trial sections should be constructed later in this project to account for the industrial manufacturing issues of WMA with lignin.

Analysis of direct costs revealed savings of 12.5% when manufacturing WMA with lignin compared to WMA with organic wax as an additive. Based on the improvement of the mechanical behaviour evaluated so far, there are expectations of reaching a 30% cost reduction, but these figures must be confirmed after the complementary laboratory and field experiments.

## Figures and Tables

**Table 1:** Physical properties of aggregates and specifications limits.

Property	Standard	Units	Gneiss 8/20	Gneiss 4/12	Sand 0/4	Filler	Limit
Flakiness index (FI)	EN 933-3 [37]	%	FI_15_	FI_15_	---	---	FI_20_
Resistance to fragmentation: Los Angeles (LA)	EN 1097-2 [38]	%	LA_20_	LA_20_	---	---	LA_30_
Resistance to wear: micro-Deval (M_DE_)	EN 1097-1 [39]	%	M_DE_10	M_DE_10	---	---	M_DE_15
Polished stone value (PSV)	EN 1097-8 [40]	%	PSV_50_	PSV_50_	---	---	PSV_50_
Water absorption (WA)	EN 1097-6 [41]	%	0.5	0.6	0.6	---	WA_24_1
Assessment of fines: methylene blue (MB_F_)	EN 933-9 [42]	g/kg	---	---	MB_F_10	MB_F_10	MB_F_10
Voids of dry compacted filler (ν)	EN 1097-4 [43]	%	---	---	---	32	ν_28/38_
Delta ring and ball (Δ_R&B_)	EN 13179-1 [44]	°C	---	---	---	14	Δ_R&B_

**Table 2:** Composition of lignin.

Components		Quantity
Glucan	g/kg TS	292
Lignin (Klason lignin)	% TS	64.6

TS—total solids.

**Table 3:** Wheel tracking test results.

Mixture		Specimen	RD_AIR_ (mm)		PRD_AIR_ (%)		WTS_AIR_ (mm/10^3^ Cycles)	
HMA	0% Lignin	No.1	3.4	3.5	8.5%	8.7%	0.137	0.130
		No.2	3.5		8.8%		0.122	
	20% Lignin	No.1	1.3	1.2	3.2%	3.0%	0.031	0.030
		No.2	1.2		2.9%		0.028	
WMA	0% Lignin	No.1	8.6	8.7	21.5%	21.7%	0.547	0.506
		No.2	8.8		21.9%		0.465	
	20% Lignin	No.1	6.3	6.8	15.7%	16.9%	0.322	0.336
		No.2	7.2		18.0%		0.350	
	Wax	No.1	7.4	7.9	18.4%	19.6%	0.382	0.401
		No.2	8.3		20.9%		0.421	

**Table 4:** Direct costs analysis.

Costs and Simple Resources			Average Unit Prices	HMA ^1^	WMA ^2^	
					Organic Wax	Lignin
				€	€	€
Direct costs	Materials	Bitumen 35/50	565 €/t	28.25	28.25	22.60
		Natural aggregates	11 €/t	10.45	10.45	10.45
		Lignin	100 €/t			1.00
		Organic wax	3200 €/t		2.40	
	Equipment ^3^	Asphalt plant	400 €/h	7.00	6.00	6.00
		Loader	60 €/h	1.05	1.05	1.05
		Lorries ^3^	60 €/h	4.50	4.50	4.50
		Paver	80 €/h	1.00	0.80	0.80
		Rollers ^3^	40 €/h	1.00	0.80	0.80
	Labour	Skilled workers	17 €/h	0.89	0.89	0.89
		Unskilled workers	10 €/h	1.40	1.40	1.40
	Other costs & contingencies (10%)			5.55	5.65	4.95
Overhead (10%)				6.11	6.22	5.44
Profit (10%)				6.72	6.84	5.99
TOTALS (€ per ton of mixture)				73.9	75.3	65.9
TOTALS (% of AC 14 surf 35/50)				98.2%	100.0%	87.5%

^1^ Hot-mix asphalt for surface layers; ^2^ Warm-mix asphalt for surface layers; ^3^ Includes fuel, drivers and maintenance.

## Data Availability

The data presented in this study are available on request from the corresponding author.
